# Supplementary material for: Synbiotics effects of d-tagatose and Lactobacillus rhamnosus GG on the inflammation and oxidative stress reaction of Gallus gallus based on the genus of cecal bacteria and their metabolites
Source: PLoS One. 2025 Jan 27;20(1):e0317825. doi: 10.1371/journal.pone.0317825 (PMC11771945; doi:10.1371/journal.pone.0317825)
Supplement: S1 Table — (DOCX) [file pone.0317825.s001.docx]

**S1 Table. Primer Sequence used to Detect Oxidative and Immune Stress Factors.**

| Gene | |  | Primer sequence (5’- 3’) | Gene ID |
| --- | --- | --- | --- | --- |
| GPX_1_ | Glutathione Peroxidase 1 | F | ACGGCTTCAAACCCAACTTCAC | 100857115 |
|  |  | R | GGTCGTCGTGCGGGAAGG |  |
| GPX_2_ | Glutathione Peroxidase 2 | F | CTGGTGGTGCTGGGCTTCC | 100857454 |
|  |  | R | TCCTCGTTGGTGCCGTTCTC |  |
| SOD_1_ | Superoxide Dismutase 1 | F | CGCTCGTAGGTGGTTGTATTGC | 395938 |
|  |  | R | TGCCTGCTGCTGGAAGTGG |  |
| SOD_3_ | Superoxide Dismutase 3 | F | CCGATCCAAGCAGCGTGTTAC | 422810 |
|  |  | R | CCATCAGTCTCATTATCAGCCATCAC |  |
| CAT | Catalase | F | AACGCTCCTTCTGCTGATTGAATAG | 423600 |
|  |  | R | GATGGTCGGAACATTGCCTACAC |  |
| GSR | Glutathione Reductase | F | TCTGCCGCTGATGCCATACC | 771783 |
|  |  | R | TGTGAGGAGCCGTGTACTTCTG |  |
| H6PD | Hexose-6-Phosphate Dehydrogenase | F | AGGCTGTGGTGCTGACTGAG | 428188 |
|  |  | R | CTGCTGATCTGCGTGGTGATG |  |
| HO-1 | Heme oxygenase-1 | F | CTCCCTTCCTCTGCTGTTGTTTG | 396287 |
|  |  | R | GTATGCCATCCTTGCTCTGTCAC |  |
| IL-1(β) | Interleukin-1, Beta | F | CACAGAGATGGCGTTCGTTCC | 395196 |
|  |  | R | TCAGAATCCAGGCGAGGCTTC |  |
| IL-2 | Interleukin 2 | F | ACTTAGTTCCATGTCAATACCGTCAC | 373958 |
|  |  | R | TAATGCTGTGCTTAGAATGCTCCTG |  |
| IL-4 | Interleukin 4 | F | TGCTCACACCTGCCTGGATG | 416330 |
|  |  | R | TGTTGTTGCTCCTCCCTCCTC |  |
| IL-6 | Interleukin 6 | F | TGCTATGTCAGAGGCGAATGTTG | 395337 |
|  |  | R | CTGCCATCTGTCACACGGTAAC |  |
| IgA | Immunoglobulin A | F | AGGTCCTCATCACTTTGCTTTCTG | 374117 |
|  |  | R | ACACGCTCCTCACCATCATCC |  |
| IgM | Immunoglobulin M | F | AGATGCCAGAGTCAATAGATGTGTTC | 101748581 |
|  |  | R | AAGCCTGCTGTTGCCTAATGTC |  |
| IgG | Immunoglobulin G | F | CTGGACAATGTGAGAGTAAGAGATGG | 424904 |
|  |  | R | CTGCGGCTTGGAGTTATGAAGAG |  |
| β-Actin | Reference Genes | F | AGATTAAGTGCTGGCTGTGAGTTG | 396526 |
|  |  | R | CGCTCCGCTACCTAATTCCTTTATC |  |
