# Supplementary material for: Synbiotics effects of d-tagatose and Lactobacillus rhamnosus GG on the inflammation and oxidative stress reaction of Gallus gallus based on the genus of cecal bacteria and their metabolites
Source: PLoS One. 2025 Jan 27;20(1):e0317825. doi: 10.1371/journal.pone.0317825 (PMC11771945; doi:10.1371/journal.pone.0317825)
Supplement: S2 Table — (DOCX) [file pone.0317825.s002.docx]

**S2 Table . Primer Sequences used to Detect the Content of Intestinal Bacteria.**

| Gene | Gene name | Primer sequence (5’- 3’) | |
| --- | --- | --- | --- |
| Lacter | Lactobacillus | F | ACAATGGACGCAAGTCTGATG |
|  |  | R | ACCGCTACACATGGAGTTCCACWGT |
| E.coli | Escherichia Coli | F | GAACGGTAACAGGAAGCAG |
|  |  | R | ATGAACAAAGGTATTAACTTTACT |
| Bifi | Bifidobacterium | F | GTCCGGTGTGAAAGTCCATC |
|  |  | R | GTAACGGCCCAGAGACCT |
