# Supplementary material for: Synbiotics effects of d-tagatose and Lactobacillus rhamnosus GG on the inflammation and oxidative stress reaction of Gallus gallus based on the genus of cecal bacteria and their metabolites
Source: PLoS One. 2025 Jan 27;20(1):e0317825. doi: 10.1371/journal.pone.0317825 (PMC11771945; doi:10.1371/journal.pone.0317825)
Supplement: Attachment — Submitted filename: PONE-D-24-49947.pdf [file pone.0317825.s003.pdf]

Manuscript Number: PONE-D-24-49947

Title: Synbiotics effects of d-tagatose and Lactobacillus rhamnosus GG on the inflammation and oxidative stress reaction of Gallus gallus based on the genus of cecal bacteria and their metabolites.

Overview and general recommendation:

This subject is important because abuse of feed supplement can cause oxidative stress and inflammatory responses in *Gallus gallus*. The current study examined the effect of d-tagatose on the probiotic properties of *L. rhamnosus* GG, *L. paracasei*, and *S. lactis*. Synbiotic composed of d-tagatose and *L. rhamnosus* GG were fed to *Gallus gallus* under oxidative stress and inflammation conditions.

I found the manuscript well written, well described and the references were new. I listed minor points below for editing.

Comments to Author:

Minor points

1- In abstract, 21line: you wrote **Abstracth**, kindly convert it to **Abstract.**

2- In abstract, 23 line and line 27: you wrote (We)!!! The rule of manuscript writing is to avoid using (We). So you should delete (**We**) and use formal academic words (**This study or The current study or The present study**). Kindly focus in this manuscript 14 times you wrote (We)!!! Convert all of them to formal academic words (**This study or The current study or The present study**).

3- In introduction, 33line: You wrote the abbreviation of the names of bacteria such as ***L. lactis and L. plantarum***; you should write the whole scientific names of them (***Lactococcus lactis*; *Lactobacillus plantarum*)** to be obvious to the reader because here (in introduction) you used these names of bacteria for the first time while in next sentences you can use the abbreviations only.

4- In materials and methods; you did not mention the period of sample collection or the period of this study!!! Kindly mention the period.

5- In materials and methods; 82 and 83 lines: you wrote **LB medium and MRS plates** you should write the whole names of the agars: **Luria Bertani (LB) agar and De Man–Rogosa–Sharpe (MRS) plates** not only the abbreviation to be obvious to the reader because here you used these names of agars for the first time while in next sentences you can use the abbreviations only.

6- In 90line; you wrote DPPH clearance, you should write the whole word **Diphenylpicrylhydrazine (DPPH)** not only the abbreviation to be obvious to the reader because here you used this word for the first time while in next sentences you can use the abbreviation.

7- I suggest to add list of abbreviations in current manuscript.

Kind regards
