# Supplementary material for: Synbiotics effects of d-tagatose and Lactobacillus rhamnosus GG on the inflammation and oxidative stress reaction of Gallus gallus based on the genus of cecal bacteria and their metabolites
Source: PLoS One. 2025 Jan 27;20(1):e0317825. doi: 10.1371/journal.pone.0317825 (PMC11771945; doi:10.1371/journal.pone.0317825)
Supplement: Attachment — Submitted filename: Response to Reviewers.docx [file pone.0317825.s004.docx]

**Response to Reviewers**

We would like to convey our sincere gratitude to the academic editor and reviewer for the insightful comments. The authors' appreciation for the academic editor and reviewers' hard work is deeply felt. These valuable insights have been of great assistance in enhancing the quality of our paper in a substantial manner.

**Response to Journal Requirements:**

1. Please ensure that your manuscript meets PLOS ONE's style requirements, including those for file naming.

→ Thanks for your suggestions, we revised the manuscript strictly in accordance with the PLOS ONE’s style requirements.

2. Please include a caption for figure 3a.

→ Thanks for your suggestions, in line 235, we added the caption for figure 3a.

3. Please include a caption for table 1.

→ Thanks for your suggestions, in line 84, 89, 154 and 160 we added the caption for Table 1-3, and Table S1 and Table S2 are in the “Supporting Information”which contain primer sequences.

4. Please remove any funding-related text from the manuscript and let us know how you would like to update your Funding Statement. Please include your amended statements within your cover letter.

→ Thanks for your suggestions, in line 451-452, we removed funding-related text from the manuscript, and the amended statements was added in cover letter.

5. Please upload the minimal anonymized data set necessary to replicate your study findings to a stable, public repository and provide us with the relevant URLs, DOIs, or accession numbers. For a list of recommended repositories. We will update your Data Availability statement on your behalf to reflect the information you provide.

→ Thanks for your suggestions, we are willing to make the data of this study public, we have stored raw data of this study in China National Center for Bioinformation (CNCB), and the link is: <https://ngdc.cncb.ac.cn/omix/preview/GBnactJE>. If this article is published, the metabolomics data located in this database will be automatically made publicly available.

6. Please include your full ethics statement in the ‘Methods’ section of your manuscript file. In your statement, please include the full name of the IRB or ethics committee who approved or waived your study, as well as whether or not you obtained informed written or verbal consent.

→ Thanks for your suggestions, in line 132-136, we added the ethical statement in the “Methods”, include full name of ethics committee who approved ours study and approval number etc.

**Response to Additional Editor Comments:**
1. Line 22: The term "Abstracth" should be corrected to "Abstract."

→ Thanks for your suggestions, in line 21, we have replaced “Abstracth” with “Abstract”.
2. Line 23: The manuscript mentions "L. paracasei" and "S. lactis" in the abstract, but the main focus seems to be on "L. rhamnosus GG." Clarify the role of these other strains.

→ Thanks for your suggestions, the significance of our study on the in vitro probiotic properties of *L. paracasei,* *S. lactis* and *L. rhamnosus GG* affected by d-tagatose in the following: screening out the best one from these lactic acid bacteria to form a synbiotic with d-tagatose, so as to conduct experiments on its in vivo probiotic properties, This is reflected in lines 76-79. As shown in line 28, we have added it to the abstract.
3. Line 27: The phrase "The results indicated that oxidative and immune stress factor gene expressions quantity in *Gallus gallus* decreased significantly after 14 days of treatment" is vague. Specify which genes were measured and how their expression was quantified.

→ Thanks for your suggestions, As shown in lines 37-40, we have added the names of the genes with changed expression levels as well as the amounts of change.
4. Line 35: The statement "Since China banned antibiotics in animal feed, the chicken mortality rate has increased from 2.8% to 4.2%" needs a citation for verification.

→ Thanks for your suggestions, in lines 51-52, this sentence has been verified by citing Reference [4].
5. Line 49: The phrase "unabsorbed d-tagatose tilized by microbial colonizing in the colon" should be corrected to "unabsorbed d-tagatose is utilized by microbes colonizing the colon."

→ Thanks for your suggestions, in line 67-68 we have made the modifications according to your requirements, replace “unabsorbed d-tagatose tilized by microbial colonizing in the colon” with “unabsorbed d-tagatose is utilized by microbes colonizing the colon”.
6. Line 56: The hypothesis "we hypothesize that tagatose’s in vivo probiotic properties remain to be elucidated" should be supported by a rationale or preliminary work.

→ Thanks for your suggestions, our previous expressions were unclear, we have modified the expression to make it more explicit. In lines 72-73, the authors of [17] only verified through RT-qPCR that d-tagatrose can upregulate the genes expression levels related to intestinal colonization and antioxidant in *L. rhamnosus GG*, but did not conduct verification by animal experiments or cell tests. And we need to verify this effect through animal experiments.
7. Line 65: The source of the seven-day-old *Gallus gallus* should be described in more detail, including any specific breed or strain information.

→ Thanks for your suggestions, in line 92, we have added the breed of *gallus gallus* as well as the reference genome sequence.
8. Line 78: The experimental group is described as containing 2% d-tagatose, but the rationale for choosing this concentration is not provided. Include justification or references.

→ Thanks for your suggestions, in 105-107 line, we have added the foundation for using this d-tagatose concentration.
9. Line 107: The ethics statement should include more details about the specific guidelines followed for animal handling and care.

→ Thanks for your suggestions, in 132-135 line, the guidelines followed by the animal experiment and its approval number have been added.
10. Line 116: The experimental design mentions "1 × 10^5 cfu/mL *L. rhamnosus GG*," but the concentration seems low for effective treatment. Provide justification or references for this dosage.

→ Thanks for your suggestions, in 142 line, we introduced references to support the concentration of *L. rhamnosus GG* used in this experiment.
11. Line 123: The experimental design figure (Fig 1) should be referenced more clearly in the text, explaining its relevance to the study.

→ Thanks for your suggestions, we have already made modifications. As shown in 138 line.
12. Line 130: The formula for weight gain rate (WGR) is missing proper formatting and should be corrected for clarity.

→ Thanks for your suggestions, in line 161-163, we have corrected the format of the formulas in this article.
13. Line 173: The results section lacks statistical analysis details. Include information on the statistical tests used and their significance levels.

→ Thanks for your suggestions, in line 216-218, we added the statistical methods for some of the experiments. In 233-234 line, we added the method of statistical tests and significance level analyses in animal experiment.

14. Line 189: The figure legend should specify the number of biological replicates used for each experiment. The axis labels in Fig 2a and 2b should include units (e.g., OD_600_, concentration). In Fig 2c and 2d, the statistical significance between groups should be indicated with asterisks or other markers.

→ Thanks for your suggestions, we have modified the formats of Fig 2a, Fig 2c and Fig 2d according to your suggestions but Fig 2b shows lactic acid bacteria against the growth of pathogenic bacteria under the influence of d-tagatose. The effect is reflected by the intuitive clear zones, and it is hard to add the units for the axis labels.
15. Line 191: The description of the weight gain rate (WGR) and specific growth rate (SGR) calculations should include the units used for each measurement.

→ Thanks for your suggestions, in line 236, we have added the units used for each measurement.
16. Figure 3 (Line 192): Line 192: The figure legend should explain the abbreviations used (e.g., WGR, SGR, FER). The units for weight gain rate (WGR) and specific growth rate (SGR) should be included in the axis labels (Fig. 3).

→ Thanks for your suggestions, in line 238-238, we have explained the abbreviations in the figure according to your suggestions. We have added the units (percentage) for WGR and SGR in the axis labels. However, FER has no unit and is a pure numerical value.
17. Line 214: The description of the bacterial genera analysis should include more details about the sequencing platform and methods used for data analysis.

→ Thanks for your suggestions, in line 260-267, we have enriched the description of bacterial genus analysis, including: the sequencing platform, the quality control of the sequenced data, and the analytical methods for the sequenced data.
18. Line 235: The metabolomics analysis section should specify the type of mass spectrometry used (e.g., LC-MS/MS) and the software used for data analysis.

→ Thanks for your suggestions, in line 285-291, we added the type of mass spectrometry and data processing method.
19. Line 274: Figure 7 The volcano plots (Fig 7a and 7b) should include labels for the most significantly altered metabolites. The heat maps (Fig 7c and 7d) should include a color scale to indicate the range of metabolite concentrations.

→ Thanks for your suggestions, we added the names of the most significantly altered metabolites in the volcano plots (Fig 7a and b). There is a color scale bar representing the concentration of metabolites on the right side of the heat maps (Fig 7c and d).
20. Line 294: The discussion mentions "Kho et al. [16]" but does not provide a full reference. Ensure all references are complete and correctly formatted.

→ Thanks for your suggestions, in line 371-372, we have changed the citation format and added the cited content.
21. Line 349: The conclusion states that synbiotics can treat inflammation and oxidative stress, but this claim needs to be tempered with limitations and suggestions for further research.
→ Thanks for your suggestions, in line 448-450, we have incorporated this sentence “Nevertheless, the mechanisms underlying the treatment of oxidative stress and inflammatory responses remain to be further investigated and delved into”, so as to temper the conclusion of the previous sentence.

**Response to Reviewer 1:**

1. Highlights should be written after abstract and before the introduction

→ Thanks for your suggestions, there is no "Highlights" in the format requirements for manuscripts by PLOS ONE, and we think the Highlights include: 1. This study evaluated the influence of d-tagatose on the in vitro probiotic characteristics of *S.lactis*, *L. rhamnosus*, and *L. paracasei*, screened the best synbiotic combination. 2. Through RT-qPCR, cecal microbiota and metabolomics analysis, the potential reasons for the treatment of *Gallus gallus*’ oxidative stress and inflammatory response by the synbiotic were explained.
2. Keywords should be written after the abstract not before

→ Thanks for your suggestions, there is no "Keywords" in the format requirements for manuscripts by PLOS ONE, and we think the keywords include: Poultry industry, Intestinal health; Probiotics; Synbiotic; Oxidative stress; Inflammatory response; Metabolomics, Intestinal flora;
3. There are no recommendations ?

→ Thanks for your suggestions, there is no " recommendations " in the format requirements for manuscripts by PLOS ONE, and our metabolomics data are stored in open databases, we hope more scholars able to join in the research so as to conduct a more systematic and in-depth study on the mechanism by which the synbiotic composed of *L. rhamnosus GG* and d-tagatose treats oxidative stress and inflammatory responses in animals.
4. There are no authors contributions ?

→ Thanks for your suggestions, in line 16-17, we added the author contributions to the cover, but the detailed author contributions (the work completed by the authors) have been uploaded to the paper submission system of PLOS ONE.
5. There are no acknowledgement ?

→ Thanks for your suggestions, in line 20, we added acknowledgement to the cover, in line 451-456, acknowledgements were also added after the conclusion.
6. There are no plan for the study area ?

→ Thanks for your suggestions, in line 132-133, we mentioned the manufacturers that supply *Gallus gallus*, in line 148-149, in 148-150, we mentioned the breeding sites of Gallus gallus, but did not mention the detailed breeding addresses, in line 148-149, we made a supplement.
7. The most descriptive methodologies are without any references--how ?

→ Thanks for your suggestions, in the experimental methods section, in line 117-119, 128, 141-142, 180-181, 183, we verified some unique experimental methods by citing reference documents.

8. What about the clinical signs of the investigated birds due to Aflatoxin

→ Thanks for your suggestions, in line 242-245, we cited some references to introduce the clinical signs of *Gallus gallus* caused by aflatoxin.

9. Which type of Aflatoxin did you use ?

→ Thanks for your suggestions, we used aflatoxin b1 for modeling and added this information in the manuscript, in line 140, we used the abbreviated name for aflatoxin b1, the abbreviation table is in line 89.
10.Aflatoxin is potent carcinogenic , at least induce necrosis not inflammation

→ Thanks for your suggestions, a small amount of aflatoxin will not cause necrosis or death of animals, and it will lead to oxidative stress and immune responses in animals, in line 41, we have proved this point by citing reference documents.
11. Why you did not do a histological sections for more confirmation

→ Thanks for your suggestions, it was our oversight, the animal carcasses have already been disposed of, thereby precluding the possibility of performing histological sections for verification.
12. What about the percentages of mortalities ?

→ Thanks for your suggestions, we have theoretical support from previous research. The use of aflatoxin B1 at this concentration will not lead to the death of *Gallus gallus*, throughout the process of animal modeling and treatment, no mortality was observed among *Gallus gallus*.
13. Introduction should be more concise

→ Thanks for your suggestions, we have simplified the “Introduction”, in line 59-60, we

simplified the description that *L. plantarum* can participate in the immune response of animals, in line 65-68 we simplified the description of d-tagatose’s prebiotic properties.
14. Results should be rewritten

→ Thanks for your suggestions, in line 203-204, 208, we simplified the names of culture media by using abbreviations; In line 233-234, we added the software used for significance level analysis; In line 242-245, we added the clinical signs of *Gallus gallus* caused by aflatoxin; In line 260 267, we added the data processing and analysis methods of intestinal bacterial genus; In line 285-291, we added the data processing and analysis methods of metabolomics; In line 307-311, 314-318, 327-337, we adjusted the content of this chapter so that it would be easier to understand.
15. Discussion should be debate the obtained results with others

→ Thanks for your suggestions, in line 401-403, 412-417, 420-422, 426-428, we added several sentences to enhance the discussion of the research results of others.
16. It is a very long paper

→ Thank you for your comment, we have made the greatest efforts to present the complete experimental process and results, so paper is long.
17. The IACUC-code should be written with M&M

→ Thanks for your suggestions, in line 132-135, we added IACUC to the manuscript.
Abstract :
18. There are no highlights

→ Thanks for your suggestions, in order to highlight the key points, in line 28, we incorporated the purpose and significance of conducting the experiment on the effect of d-tagatose on the in vitro probiotics characteristics, in line 37-40, we added the genes names related to oxidative stress and inflammatory responses and the changes of gene expression levels after treatment, in line 45-47, we emphasized the conclusion of this study.
19. Abstract should contain --backgrounds/methods/results and conclusion

→ Thanks for your suggestions, we altered abstract’s structure, include three parts of backgrounds, methods, results and conclusion.
20. Abstract is very short

→ Thanks for your suggestions, in order to expand abstract’s content, in line 28, we incorporated the purpose and significance of conducting the experiment on the effect of d-tagatose on the in vitro probiotics characteristics, in line 37-40, we added the genes names related to oxidative stress and inflammatory responses and the changes of gene expression levels after treatment.
21. LN/24--what is /are the differences between probiotics/prebiotics and synbiotics (in table---role/mechanism --etc--with reference ) ???

→ Thanks for your suggestions, references and tables should’t be included in the abstract according to the format requirements of PLOS ONE, therefor, in line 84, we included the differences and connections among probiotics, prebiotics and synbiotics and cited the reference literature.
22. LN/25--inflammatory conditions---mention all

→ Thanks for your suggestions, in line 30, we described the formative reasons of inflammation in gallus gallus - “Oxidative stress and immune response caused by aflatoxin B1.”
23. LN/26--rewrite again (should write oxidative stress and immune response --etc )

→ Thanks for your suggestions, in line 29-30, we have rewritten the part about "oxidative stress and immune response according to your suggestions.
24. LN/32--add poultry industry / intestinal health /probiotics to the keywords

→ Thanks for your suggestions, there is no "Keywords" in the format requirements for manuscripts by PLOS ONE, and we added the keywords of “Poultry industry” “Intestinal” “Probiotics” in paper submission system.
Introduction
25. Introduction should be more concise

→ Thanks for your suggestions, we have simplified the “Introduction”, in line 59-60, we

simplified the description that *L. plantarum* can participate in the immune response of animals, in line 65-68 we simplified the description of d-tagatose’s prebiotic properties.
26. Aims need to be more clarified

→ Thanks for your suggestions, in line 76-83, we have rewritten this paragraph so that the goal is more definite.
27. Novelty of this study should be more highlighted and more adjusted

→ Thanks for your suggestions, in line 76-83, the last paragraph in the Introduction has been modified to better highlight the novelty of this study.
Materials and methods :
28. There are no plan for the study area

→ Thanks for your suggestions, in line 132-133, we mentioned the manufacturers that supply *Gallus gallus*, in line 148-149, in 148-150, we mentioned the breeding sites of Gallus gallus, but did not mention the detailed breeding addresses, in line 148-149, we made a supplement.
29. The most descriptive methods are without references --why ???

→ Thanks for your suggestions, in the experimental methods section, in line 117-119, 128, 141-142, 180-181, 183, we verified some unique experimental methods by citing reference documents.
30. What about the LD50 of Aflatoxin-----?

→ Thanks for your suggestions, taking rats as an example, LD50 of Aflatoxin is 0.48 mg/kg, In this study, only 50 micrograms of aflatoxin B1 was added to 1 kilogram of feed. The content was extremely low and would not lead to the death of *Gallus gallus*. However, it will cause oxidative stress and immune responses.
31. LN/65--delete experimental

→ Thanks for your suggestions, in line 90 we deleted the “experimental” in “experimental materials”.
32. LN/66-70---what about systems of housing (watering/lighting--etc)

→ Thanks for your suggestions, in line 148-149, we have already described the systems of housing.
33. Aflatoxin is potent carcinogenic not inflammation induce, at least induce necrosis

→ Thanks for your suggestions, a small amount of aflatoxin will not cause necrosis or death of animals, and it will lead to oxidative stress and immune responses in animals, in line 134, we proved this point by citing reference documents.
34. LN/107--write IACUC code --should be

→ Thanks for your suggestions, in line 126-130, we added IACUC to the manuscript.
35. All experimental groups should be tabulated and done according to whom !!!

→ Thanks for your suggestions, in line 148, we added the Tbale 2 to describe the grouping situation.
Results
36. All descriptions under figures should be more summarize

→ Thanks for your suggestions, in line 217,221, 242, 294, 320, 325, 331, we have tried our best to simplify the description under the figure.
37. Should be rewritten again

→ Thanks for your suggestions, in line 203-204, 208, we simplified the names of culture media by using abbreviations; In line 233-234, we added the software used for significance level analysis; In line 242-245, we added the clinical signs of *Gallus gallus* caused by aflatoxin; In line 260 267, we added the data processing and analysis methods of intestinal bacterial genus; In line 285-291, we added the data processing and analysis methods of metabolomics; In line 307-311, 314-318, 327-337, we adjusted the content of this chapter so that it would be easier to understand.
38. What about the clinical signs of the investigated birds due to Aflatoxin

→ Thanks for your suggestions, in line 228-231, we added the clinical signs of *gallus gallus* influenced by aflatoxin b1.
39. Aflatoxin is potent carcinogenic , at least induce necrosis not inflammation

→ Thanks for your suggestions, a small amount of aflatoxin will not cause necrosis or death of animals, and it will lead to oxidative stress and immune responses in animals, in line 134, we proved this point by citing reference documents.
40. Why you did not do a histological sections for more confirmation

→ Thanks for your suggestions, it was our oversight, the animal carcasses have already been disposed of, thereby precluding the possibility of performing histological sections for verification.
41. What about the percentages of mortalities ?

→ Thanks for your suggestions, we have theoretical support from previous research. The use of aflatoxin b1 at this concentration will not lead to the death of *Gallus gallus*.
Discussion :
42. Should be based upon debating the obtained results with those of the previous investigators

→ Thanks for your suggestions, in line 401-403, 412-417, 420-422, 426-428, we added several sentences to enhance the discussion of the research results of others.
References :
43. Some cited references need to be more updated

→ Thanks for your suggestions, we have updated references [2, 10]. Almost all of the references are within five years.
44. As volume/issue/pages/number--available----so no need for the link(s)---apply for all

→ Thanks for your suggestions, we have removed the links to literature citations in the main text.
**Response to Reviewer 2:**
1. In abstract, 21line: you wrote Abstracth, kindly convert it to Abstract.

→ Thanks for your suggestions, in line 21, we replaced “Abstracth” with “Abstract”.
2. In abstract, 23 line and line 27: you wrote (We)!!! The rule of manuscript writing is to avoid using (We). So you should delete (We) and use formal academic words (This study or The current study or The present study). Kindly focus in this manuscript 14 times you wrote (We)!!! Convert all of them to formal academic words (This study or The current study or The present study).

→ Thanks for your suggestions, in order to make the vocabulary more formal, we replace “we” with formal academic vocabulary in ours manuscript.
3. In introduction, 33line: You wrote the abbreviation of the names of bacteria such as L. lactis and L. plantarum; you should write the whole scientific names of them (Lactococcus lactis; Lactobacillus plantarum) to be obvious to the reader because here (in introduction) you used these names of bacteria for the first time while in next sentences you can use the abbreviations only.

→ Thanks for your suggestions, in line 89, we added a list that includes the abbreviations of bacteria names.

4. In materials and methods; you did not mention the period of sample collection or the period of this study!!! Kindly mention the period.

→ Thanks for your suggestions, in line 150-152, we added the period of animal treatment and the time of sample collection in "Design of the animal experiments".
5. In materials and methods; 82 and 83 lines: you wrote LB medium and MRS plates you should write the whole names of the agars: Luria Bertani (LB) agar and De Man–Rogosa–Sharpe (MRS) plates not only the abbreviation to be obvious to the reader because here you used these names of agars for the first time while in next sentences you can use the abbreviations only.

→ Thanks for your suggestions, in line 89, we added a list that includes the abbreviations of the culture media names. In line 103, we replaced “LB medium” with “LB broth” so as to express more precisely, in line 111-112, we rewrote “MRS plates” and “MRS solid medium” into the names in the abbreviation table such as “MRS-Tag agar”
6. In 90line; you wrote DPPH clearance, you should write the whole word Diphenylpicrylhydrazine (DPPH) not only the abbreviation to be obvious to the reader because here you used this word for the first time while in next sentences you can use the abbreviation.

→ Thanks for your suggestions, in line 89, we added a list that includes the abbreviations of free radical names, this can not only make the content of the article more organized, but also make it easier for readers to understand.
7. I suggest to add list of abbreviations in current manuscript.
→ Thanks for your suggestions, this suggestion has been of great help to us! In line 89, we added a list of abbreviations so as to make it easier for readers to understand the names of our bacteria, reagents, and culture media.
